# DEB‐TACE versus cTACE for unresectable HCC with B1‐type bile duct invasion after successful biliary drainage: A propensity score matching analysis

**DOI:** 10.1002/cam4.7419

**Published:** 2024-07-06

**Authors:** Wenzhe Fan, Xinlin Zheng, Xiao Zhao, Bowen Zhu, Yanqin Wu, Miao Xue, Rong Tang, Zhen Huang, Liangliang Qiao, Mingjian Lu, Yiyang Tang, Jian Wu, Jiaping Li

**Affiliations:** ^1^ Department of Interventional Oncology The First Affiliated Hospital of Sun Yat‐sen University Guangzhou China; ^2^ Cancer Center The First Affiliated Hospital of Sun Yat‐sen University Guangzhou China; ^3^ Department of Hepatopancreatobiliary Surgery Hainan General Hospital Haikou China; ^4^ Department of Interventional Angiology Huizhou First People's Hospital Huizhou China; ^5^ Department of Interventional Oncology Jinshazhou Hospital of Guangzhou University of Chinese Medicine Guangzhou China; ^6^ Department of Radiology Affiliated Cancer Hospital & Institute of Guangzhou Medical University Guangzhou China; ^7^ Center of Hepato‐Pancreato‐Biliary Surgery The First Affiliated Hospital of Sun Yat‐sen University Guangzhou China

**Keywords:** bile duct invasion, hepatocellular carcinoma, safety, transarterial chemoembolization, treatment outcome

## Abstract

**Background:**

Transarterial chemoembolization (TACE) is the standard treatment for intermediate‐stage hepatocellular carcinoma (HCC). Given the lack of specific recommendations for conventional TACE (cTACE) and drug‐eluting bead TACE (DEB‐TACE) in patients having unresectable HCC with tumor infiltrating the common hepatic duct or the first‐order branch of the bile ducts (B1‐type bile duct invasion; B1‐BDI) after biliary drainage, we retrospectively compared the safety and efficacy of DEB‐TACE with cTACE in this patient population.

**Materials and Methods:**

Using data from five tertiary medical centers (January 2017–December 2021), we compared complications, overall survival (OS), time to progression (TTP), and tumor response rate between patients having unresectable HCC with B1‐BDI who underwent DEB‐TACE or cTACE after successful biliary drainage. X‐tile software calculated the pre‐TACE total bilirubin (TBil) cutoff value, indicating optimal timing for sequential TACE after drainage. Propensity score matching (PSM) was performed.

**Results:**

The study included 108 patients with unresectable HCC (B1‐BDI) who underwent DEB‐TACE and 114 who received cTACE as initial treatment. After PSM (*n* = 53 for each group), the DEB‐TACE group had a longer TTP (8.9 vs. 6.7 months, *p* = 0.038) and higher objective response rate (64.2% vs. 39.6%, *p* = 0.011) than did the cTACE group, although OS was comparable (16.7 vs. 15.3 months, *p* = 0.115). The DEB‐TACE group exhibited fewer post‐procedural increments in the mean albumin‐bilirubin score, TBil, and alanine aminotransferase (ALT), along with a significantly lower incidence of serious adverse events within 30 days (hepatic failure, ALT increase, and TBil increase) than the cTACE group (all *p* < 0.05). The pre‐TACE TBil cutoff value was 99 μmol/L; patients with higher values (>99 μmol/L) had poorer OS in both groups (*p* < 0.05).

**Conclusion:**

DEB‐TACE is safe and effective after successful biliary drainage in unresectable HCC with B1‐BDI, potentially better than cTACE in terms of liver toxicity, TTP, and ORR. Lowering TBil below 99 μmol/L through successful drainage may create ideal conditions for sequential TACE.

## 1 INTRODUCTION

Bile duct invasion (BDI) by hepatocellular carcinoma (HCC), occurring in 0.53%–12.9% of cases,^1^, ^2^ is termed “icteric‐type hepatoma” due to its presentation with obstructive jaundice caused by tumor infiltration or external compression of the bile duct.^3^, ^4^ HCC patients with BDI have a poor prognosis owing to jaundice‐induced impairment and hepatic failure, and treatment strategies are poorly defined in current guidelines.^5^, ^6^ HCC‐related BDI is classified as B1 (central type: invasion of the common hepatic duct or first‐order branch of bile ducts with or without microscopic invasion of the intrahepatic peripheral bile duct) or B2 (peripheral type: invasion of second‐order or higher peripheral branches of the bile duct but no invasion of the first‐order branch or common hepatic duct).^7^ Typically, B2‐BDI is detected only through postoperative pathological examinations, with surgical treatment considered a radical therapeutic option.^7^ However, B1‐BDI can be identified by abnormally elevated serum bilirubin levels and biliary dilation above the obstruction on imaging.^1^ Most patients with B1‐BDI HCC are not eligible for surgery due to poor liver function or advanced tumor disease. Furthermore, the risk of intrahepatic recurrence after surgery is high owing to its infiltrative nature.^8^ In these inoperable patients, biliary drainage can alleviate obstructive jaundice, facilitating further management.^9^ Nonetheless, the optimal post‐drainage antitumor treatments remain unclear.

HCC patients with BDI who receive only palliative treatment have a dismal prognosis, with a 1‐year survival rate of 0%.^10^, ^11^ However, successful biliary drainage followed by treatments like conventional transarterial chemoembolization (cTACE) can improve the 1‐year survival rate to 50.9%.^12^ TACE, including cTACE and TACE with drug‐eluting beads (DEB‐TACE), is the standard local therapy for unresectable intermediate‐stage HCC.^13^ cTACE involves injecting a mixture of lipiodol‐based emulsion, embolizing agents, and cytotoxic drugs into the hepatic feeding artery for HCC treatment.^14^ Despite its efficacy, cTACE has limitations due to the mobility of lipiodol and uncontrolled drug release, leading to issues like unstable embolization effects, brief drug concentration in the target lesion,^15^ and potential systemic adverse effects.^16^ To overcome these limitations, an advanced drug delivery system, DEB‐TACE, was developed. It employs microspheres as embolic agents loaded with water‐soluble drugs for precise and sustained delivery to the target lesion without highly increasing systemic drug levels.^17^ However, clear guidelines for selecting between cTACE or DEB‐TACE in unresectable HCC cases are lacking.^18^ The PRECISION V Study has demonstrated that DEB‐TACE outperforms cTACE in advanced HCC, affording higher objective response rates (ORR), and lower liver toxicity.^19^, ^20^ Unresectable HCC with BDI is commonly accompanied by fragile liver function and heavy tumor burden^12^, ^21^; hence, DEB‐TACE is theoretically a more suitable approach after successful drainage.

Currently, there is a lack of studies on using DEB‐TACE in these patients, especially in comparison with cTACE. Thus, we retrospectively analyzed data from HCC patients with B1‐BDI who underwent either DEB‐TACE or cTACE after biliary drainage. Our aim was to compare their outcomes and adverse effects, and to find the best timing for TACE after successful drainage based on pre‐TACE total bilirubin (TBil) levels.

## 2 METHODS

### 2.1 Study population

The study was approved by the relevant Institutional Review Board, and the need for informed consent was waived owing to the retrospective nature of the study. All procedures involving human participants were conducted in accordance with the Declaration of Helsinki. This retrospective study used data collected at five tertiary medical centers from January 1, 2017, to December 30, 2021.

Eligibility criteria were as follows: (a) 18–75 years; (b) unresectable HCC diagnosed according to the American Association for Liver Diseases and European/American Association for Liver Diseases^5^, ^6^; (c) at least one typical enhanced measurable intrahepatic target lesion based on the modified Response Evaluation Criteria in Solid Tumors (mRECIST) criteria^22^; (d) B1‐BDI diagnosis based on elevated TBil level and the typical appearance of cholangiectasis (common duct diameter >6 mm and intrahepatic duct diameter >3 mm) caused by a tumor infiltrating the common hepatic duct or the first‐order branch of the bile ducts, as observed in CT or MRI^1^, ^7^; (e) successful bile duct drainage was achieved by percutaneous transhepatic cholangial drainage (PTCD) or stent implantation through PTCD or endoscopic retrograde cholangiopancreatography (ERCP) before TACE; and (f) TACE was performed as the first‐line local treatment for HCC after successful bile duct drainage.

**FIGURE 1:**
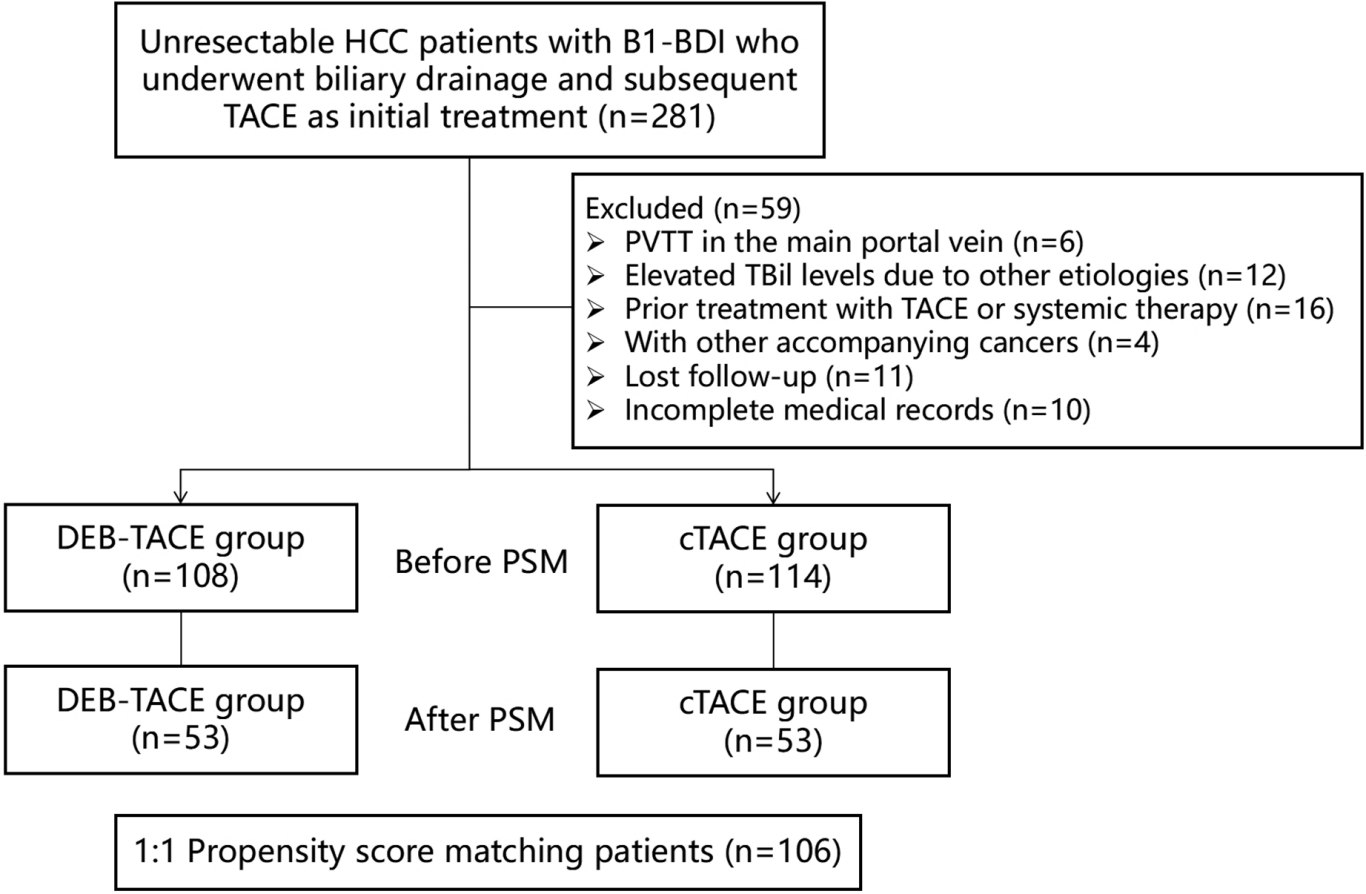
Patient inclusion flowchart. A total of 281 patients received biliary drainage and subsequent TACE as an initial treatment for unresectable HCC with B1‐BDI between January 1st 2017 and December 30th 2021. Among them, 108 patients in the DEB‐TACE group and 114 in the cTACE group met the eligibility criteria. Performing PSM resulted in matched cohorts of 53 patients per group with well‐balanced baseline characteristics. BDI, bile duct invasion; cTACE, conventional transarterial chemoembolization; DEB‐TACE, drug‐eluting beads transarterial chemoembolization; HCC, hepatocellular carcinoma; PSM, propensity score matching; PVTT, portal vein tumor thrombosis.

Patients who met the following criteria were excluded: (a) portal vein tumor thrombus (PVTT) in the main portal vein; (b) elevated TBil levels due to other etiologies; (c) prior treatment with TACE or systemic therapy; (d) presence of other accompanying cancers; (e) loss to follow‐up; and (f) incomplete medical records. Subsequently, 281 patients with unresectable HCC who presented with B1‐BDI‐induced symptomatic obstructive jaundice at diagnosis were enrolled (Figure 1).

### 2.2 Successful biliary drainage

After diagnosing HCC with B1‐BDI presenting obstructive jaundice, biliary drainage was performed either by PTCD or biliary stenting using PTCD/ERCP. Cholangitis was prevented or managed by antibiotics before TACE. Successful biliary drainage is defined as a reduction in the serum TBil concentration to >50% of the pre‐procedural value or a decrease to <3 mg/dL within 4 weeks and the absence of cholangitis.^23^ TACE was administered after successful drainage was identified, and the patient's general condition was conducive for the procedure. The pre‐TACE TBil level was the TBil level measured within 3 days before TACE after successful biliary drainage had been achieved.

### 2.3 TACE procedures

TACE after successful bile duct drainage was performed by interventional radiologists with at least 5 years of experience. Standard angiographic facilities and protocols were employed for hepatic angiography and catheterization. In all patients, the celia and superior mesenteric arteries were imaged to evaluate the liver vasculature prior to treatment. Super‐selective catheterizations by microcatheters were performed in every DEB‐TACE or cTACE procedure whenever possible. However, in patients with bilobar multinodular disease, lobar artery was selective to catheterize at least.

For cTACE, a water‐in‐oil‐type emulsion^16^ containing 50 mg of doxorubicin (Adriamycin; Pharmacia & Upjohn) with 10–20 mL of lipiodol (Guerbet, Villepinte) was infused, followed by 150–350 μm of gelatin sponge particles (Gelatin Sponge Particle Embolic Agent; Alicon) until stasis was almost attained. For DEB‐TACE, DEB was used as recommended.^24^ CalliSpheres microspheres (Jiangsu Hengrui Medical Co., Ltd.) or DC Beads (Biocompatibles UK), measuring 100–300 or 300–500 μm, were employed. Subsequently, 75 mg doxorubicin in sterile water was loaded into each DEB vial (2 mL of beads). The DEB dose was determined by tumor volume (calculation of ellipsoid volume: height × width × length × π/6). To prevent doxorubicin overload in doxorubicin‐loaded drug‐eluting beads, embolization protocols followed in our research rarely necessitated supplementary embolic materials, and complete devascularization required 300–500 μm Embosphere® microspheres (Merit Medical System Inc, USA).^24^ The primary chemoembolization endpoint was complete devascularization of the HCC observed on the angiography.^25^ TACE procedures were repeated on demand at an interval of 4–6 weeks upon identification of viable tumors or intrahepatic recurrences by CT/MRI in patients with the appropriate clinical and laboratory findings. Repeated TACE treatment was continued unless there was unacceptable toxicity or progression meeting the TACE refractoriness criteria,^26^ established by the Japan Society of Hepatology.

### 2.4 Assessment of outcomes and adverse events

**FIGURE 2:**
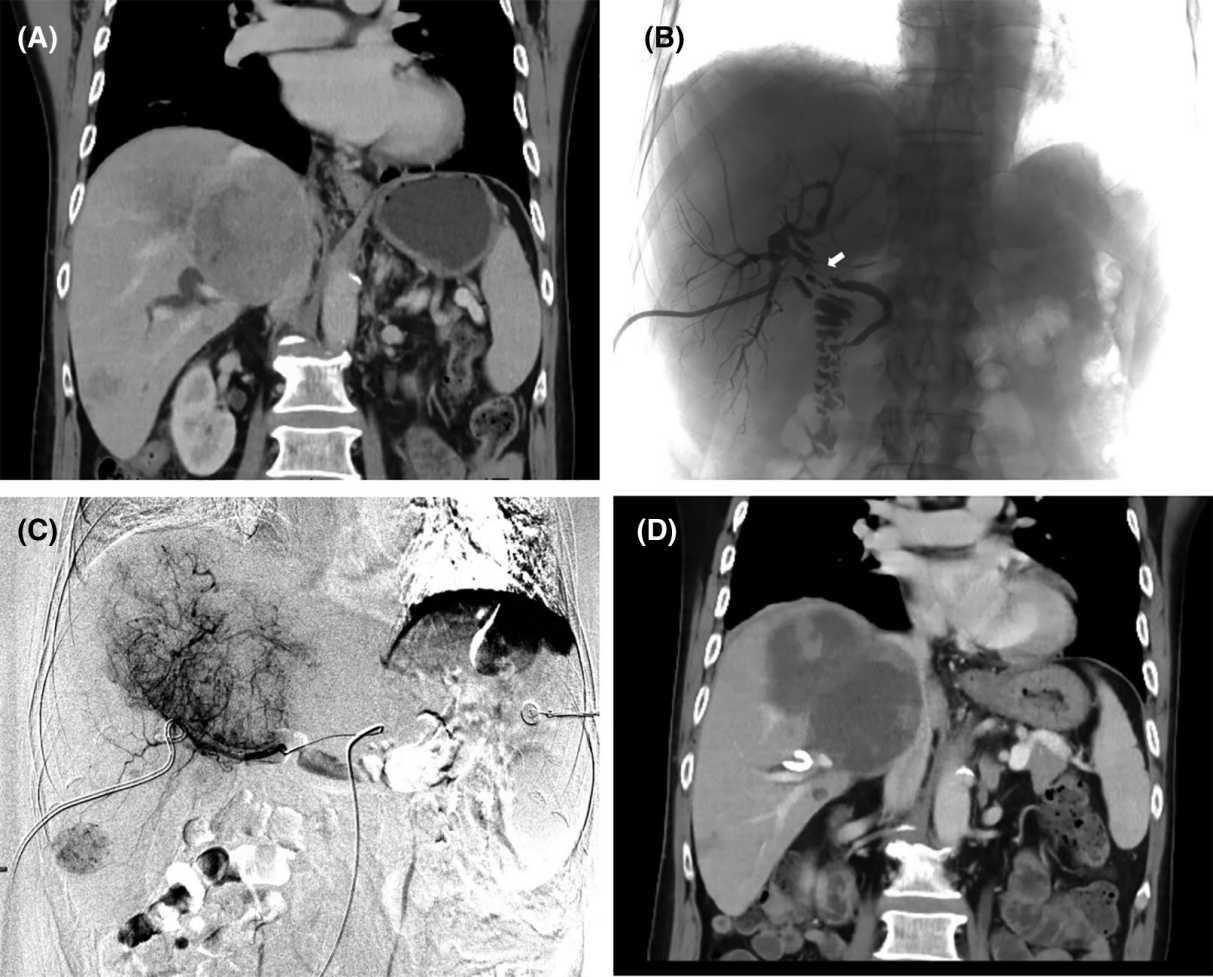
A 67‐year‐old man with B1‐BDI unresectable hepatocellular carcinoma: (A) Enhanced CT at diagnosis shows multiple nodular lesions and bile duct dilation in liver; (B) Percutaneous transhepatic cholangial drainage (PTCD) was performed with one external drainage tubes. Under digital subtraction angiography monitoring, imaging showed a filling defect inside the bile duct (white arrow); (C) The diagnostic arteriography revealed hypervascular lesions during the first DEB‐TACE procedure; (D) Enhanced CT at 4 weeks after successful drainage by PTCD and sequential DEB‐TACE shows obvious necrosis in the intrahepatic tumors. cTACE, conventional transarterial chemoembolization; DEB‐TACE, drug‐eluting beads transarterial chemoembolization.

Overall survival (OS) was measured from the first TACE after successful bile duct drainage to death or the last follow‐up. Time to progression (TTP) was defined as the number of days from the first TACE until the detection of progressive disease. All patients underwent enhanced CT or MRI within 1 week prior to the treatment and assessment of laboratory parameters, including serum alpha‐fetoprotein (AFP) level and hepatic function parameters, such as serum albumin (ALB) and TBil levels, within 72 h before, 1 week after, and 1 month after every TACE. Tumor response and safety were assessed at 1‐month intervals until death or the endpoint. Residual tumor or recurrence was treated by performing additional TACE according to the above‐described criteria. Only one of the two TACE approaches was allowed during repeated TACE procedures before progressive disease. The efficacy of local tumor response after TACE, classified as complete response (CR), partial response (PR), stable disease (SD), or progressive disease (PD), was assessed using mRECIST criteria. The disease control rate (DCR) was the sum of the CR, PR, and SD rates; the ORR was the sum of CR and PR rates.^22^ The best overall response during treatment was considered the final response and was recorded. Target lesion responses were assessed by two independent radiologists blinded to respective evaluations; any inconsistencies were resolved by discussion. The primary endpoint was OS. The secondary endpoints were TTP, treatment response and adverse event. The last follow‐up date was August 1, 2022. Figure 2 presents a typical case.

Adverse events were classified according to the Common Terminology Criteria of Adverse Events (CTCAE) v4.0.^27^ Serious adverse events were documented if National Cancer Institute (NCI) CTCAE grade 3 or 4 laboratory values or clinical states were observed within 1 month after TACE. The hepatic reserve function was evaluated according to TBil and ALB levels and the ALB‐bilirubin (ALBI) score. The ALBI score was calculated as follows: ALBI score = (−0.085 × ALB [g/L]) + (0.66 × log_10_ TBil [μmol/L]).^28^

### 2.5 Statistical analysis

Comparisons between groups were performed using Student's *t*‐test for continuous variables, expressed as mean ± standard deviation, and Pearson's chi‐squared (χ^2^) test for categorical data, presented as frequency values. Survival curves were assessed using Kaplan–Meier analyses, with univariate analysis performed using the log‐rank test. Multivariate analysis was performed using Cox regression analysis for variables that showed *p* < 0.05 in the univariate analysis.

Propensity score matching (PSM) analysis was used to reduce the possibility of selection bias from an imbalance in variables that could potentially influence outcomes. The propensity score model included the following variables: age, sex, presence of hepatitis B virus, AFP, ALB, TBil levels on admission and before TACE, intrahepatic tumor number, tumor size, PVTT, extrahepatic metastasis, alanine aminotransferase (ALT) level, and Barcelona Clinic Liver Cancer (BCLC) stage. PSM was performed using a 1:1 matching method with a caliper width of 0.1 times the standard deviation. All statistical tests were two‐sided, and *p* < 0.05 indicated statistical significance. Programming and statistical analyses were performed using R version 4.0.2 (Stanford University, CA, USA) and STATA version 15.0 software (Stata Corporation, College Station, TX, USA).

We also used X‐tile 3.61 (Yale University, New Haven, CT, USA)^29^ to determine the optimal cutoff pre‐TACE TBil level based on the OS; this step facilitated the selection of the optimal time to perform TACE after bile duct drainage for patients with unresectable HCC presenting with B1‐BDI (before PSM, to approximate real‐world treatment conditions). Kaplan–Meier curve analysis and univariate Cox regression analysis were performed to confirm the accuracy of the cutoff pre‐TACE TBil in the DEB‐TACE and cTACE groups, respectively.

## 3 RESULTS

### 3.1 Baseline characteristics

**FIGURE 3:**
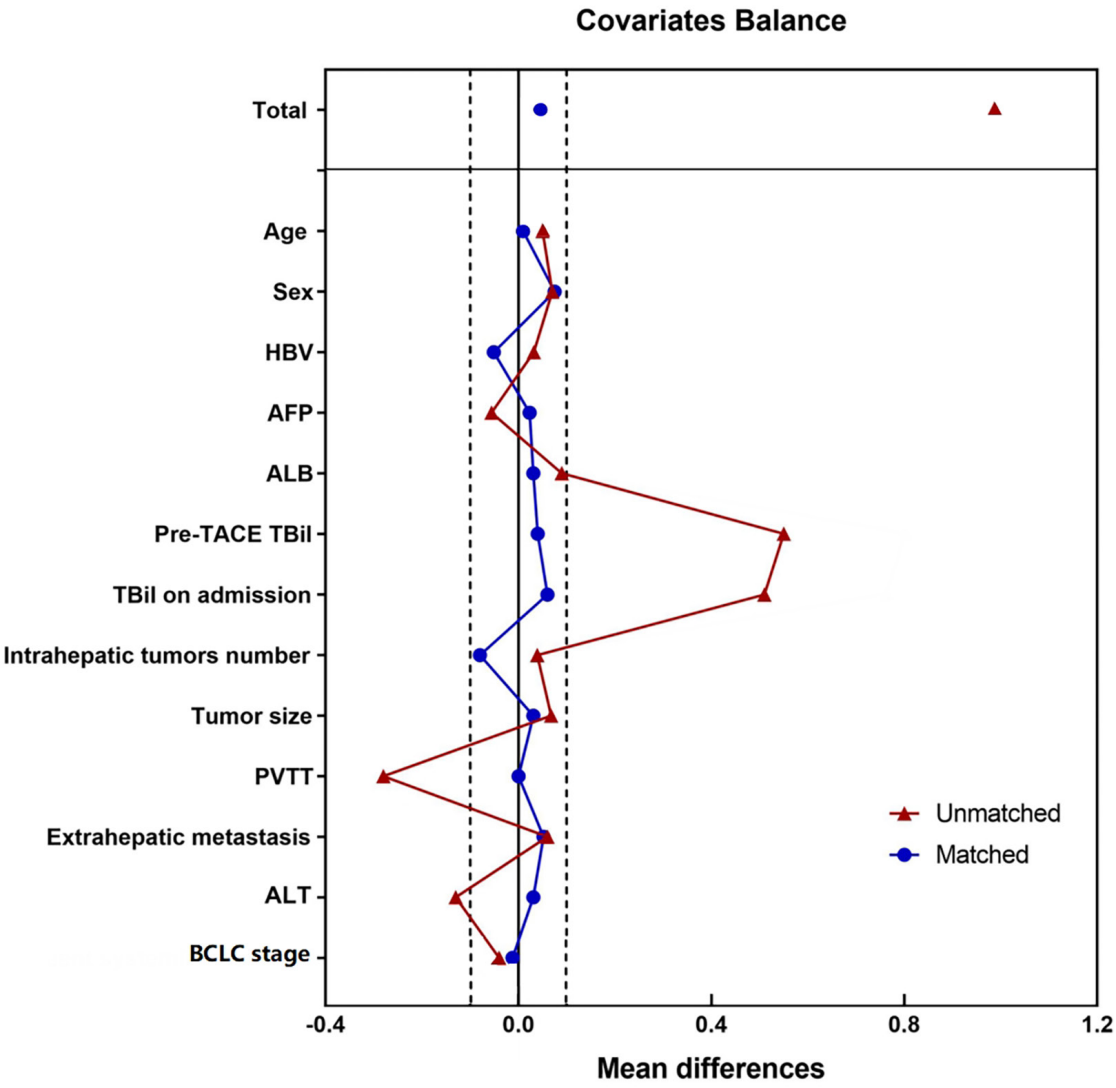
Mean differences in covariates between unresectable HCC patients with B1‐BDI receiving DEB‐TACE or cTACE before and after propensity score‐matching analysis.

Before PSM, 222 patients (DEB‐TACE, *n* = 108; cTACE, *n* = 114) were included, and the cTACE group had higher ALT levels (*p* = 0.007), TBil levels on admission (*p* < 0.001), pre‐TACE TBil levels (*p* < 0.001), and ALBI scores (*p* = 0.001) and more patients with PVTT (*p* = 0.032) than the DEB‐TACE group. After PSM, matched cohorts of 53 patients with well‐balanced baseline characteristics were extracted from each group. The baseline clinicopathological characteristics of both groups are shown in Figure 1 and Table 1. The covariate balance between the two groups before and after PSM was assessed and represented in a love plot (Figure 3).

**TABLE 1:** Baseline characteristics.

Characteristics	Before PSM			After PSM		
	Number (%)/Median (IQR)^a^					
	DEB‐TACE group (*n* = 108)	cTACE group (*n* = 114)	*p* Value	DEB‐TACE group (*n* = 53)	cTACE Group (*n* = 53)	*p* Value
Age (y)	54.2 ± 11.5	54.6 ± 10.2	0.764	53.6 ± 11.5	55.4 ± 9.5	0.813
<50	39 (36.1%)	38 (33.3%)		19 (35.9%)	15 (28.3%)	
≥50	69 (63.9%)	76 (66.7%)		34 (64.2%)	38 (71.7%)	
Sex						
Male	91 (84.3%)	102 (89.5%)	0.249	46 (86.8%)	50 (94.3%)	0.319
Female	17 (15.7%)	12 (10.5%)		7 (13.2%)	3 (5.7%)	
HBV						
Absence	19 (17.6%)	21 (18.4%)	0.872	7 (13.2%)	10 (18.9%)	0.427
Presence	89 (82.4%)	93 (81.6%)		46 (86.8%)	43 (81.1%)	
AFP (ng/mL)						
<200	57 (52.8%)	69 (60.5%)	0.244	31 (58.5%)	30 (56.6%)	0.844
≥200	51 (47.2%)	45 (39.5%)		22 (41.5%)	23 (43.4%)	
ALB (g/L)	35.2 (34.1–36.2)	33.9 (33.0–34.7)	0.052	34.2 (32.8–35.5)	34.7 (33.5–35.9)	0.733
ALT (U/L)	62.9 (49.0–76.8)	89.6 (75.9–103.3)	0.007	67.6 (47.7–87.5)	80.7 (62.8–98.5)	0.329
TBil on admission^b^ (umol/L)	167.4 (146.8–187.9)	227.6 (208.7–246.5)	<0.001	179.4 (151.7–206.9)	188.0 (163.3–212.7)	0.641
Pre‐TACE TBil^c^ (umol/L)	75.5 (66.8–84.1)	98.4 (89.8–107.1)	<0.001	82.6 (69.1–96.1)	84.7 (73.0–96.5)	0.810
Intrahepatic tumors number						
1	61 (56.5%)	66 (57.9%)	0.832	32 (60.4%)	25 (47.2%)	0.173
≥2	47 (43.5%)	48 (42.1%)		21 (39.6%)	28 (52.8%)	
Tumor size^d^ (cm)	7.04 (6.26–7.83)	6.49 (5.60–7.37)	0.357	7.18 (6.08–8.28)	6.94 (5.56–8.32)	0.788
<5 cm	38 (35.2%)	61 (53.5%)		18 (34.0%)	26 (49.1%)	
≥5 cm	70 (64.8%)	53 (46.5%)		35 (66.0%)	27 (50.9%)	
PVTT						
Absence	61 (56.5%)	48 (42.1%)	0.032	23 (43.4%)	23 (43.4%)	1.000
Presence	47 (43.5%)	66 (57.9%)		30 (56.6%)	30 (56.6%)	
Biliary drainage						
PTCD	97 (89.8%)	98 (86.0%)	0.380	49 (92.5%)	47 (88.7%)	0.506
Biliary stent	11 (10.2%)	16 (14.0%)		4 (7.5%)	6 (11.3%)	
Extrahepatic metastasis						
Absence	80 (74.1%)	78 (68.4%)	0.353	40 (75.5%)	36 (67.9%)	0.388
Presence	28 (25.9%)	36 (31.6%)		13 (24.5%)	17 (32.1%)	
ALBI score	−1.81 [(−1.91) to (−1.70)]	−1.59 [(−1.67) to (−1.52)]	0.001	−1.69 [(−1.82) to (−1.56)]	−1.72 [(−1.83) to (−1.61)]	0.711
BCLC stage						
A	15 (13.9%)	8 (7.0%)	0.061	4 (7.6%)	6 (11.3%)	0.808
B	46 (42.6%)	40 (35.1%)		19 (35.9%)	17 (32.1%)	
C	47 (43.5%)	66 (57.9%)		30 (56.6%)	30 (56.6%)	

Abbreviations: AFP, alpha‐fetoprotein; ALB, albumin; ALT, alanine aminotransferase; ALBI, albumin‐bilirubin; BCLC, Barcelona Clinic Liver Cancer; cTACE, conventional transarterial chemoembolization; DEB‐TACE, drug‐eluting beads transarterial chemoembolization; PSM, propensity score matching; PTCD, percutaneous transhepatic cholangial drainage; PVTT, portal vein tumor thrombus; TBil, total bilirubin.

^a^ Median with interquartile range is shown for quantitative variables, whereas counts with proportions are shown for categorical variables.

^b^ TBil, TBil measured at the time of the first diagnosis.

^c^ Pre‐TACE TBil, TBil measured after successful drainage before performing TACE.

^d^ Tumor size, size of the largest tumor.

### 3.2 Treatment outcomes

Median interval between biliary drainage and TACE was 6.8 days (range 1–30 days). After PSM, according to mRECIST criteria, 4 (7.5%), 30 (56.6%), 11 (20.8%), and 8 (15.1%) patients in the DEB‐TACE group and 2 (3.8%), 19 (35.8%), 16 (30.2%), and 16 (30.2%) in the cTACE group achieved CR, PR, SD, and PD, respectively. The DEB‐TACE group showed higher PR (56.6% vs. 35.8%, *p* = 0.032) and ORR (64.2% vs. 39.6%, *p* = 0.011) than the cTACE group, with similar DCR (84.9% vs. 69.8%, *p* = 0.063) (Table 2).

**TABLE 2:** Tumor response^a^ in patients between the two groups (after propensity score matching).

Response category	DEB‐TACE (*n* = 53) (%)	cTACE (*n* = 53) (%)	*p* Value
CR	4 (7.5%)	2 (3.8%)	0.678
PR	30 (56.6%)	19 (35.8%)	0.032
ORR (CR + PR)	34 (64.2%)	21 (39.6%)	0.011
DCR (CR + PR + SD)	45 (84.9%)	37 (69.8%)	0.063

Abbreviations: CR, complete response; cTACE, conventional transarterial chemoembolization; DCR, disease control rate, DCR = CR + PR + SD; DEB‐TACE, drug‐elutingbeads transarterial chemoembolization; mRECIST, modified Response Evaluation Criteria in Solid Tumors; ORR, objective response rate, ORR = CR + PR; PR, partial response; SD, stable disease.

^a^ The response assessment of the DEB‐TACE and cTACE groups analyzed according to the mRECIST criteria.

**FIGURE 4:**
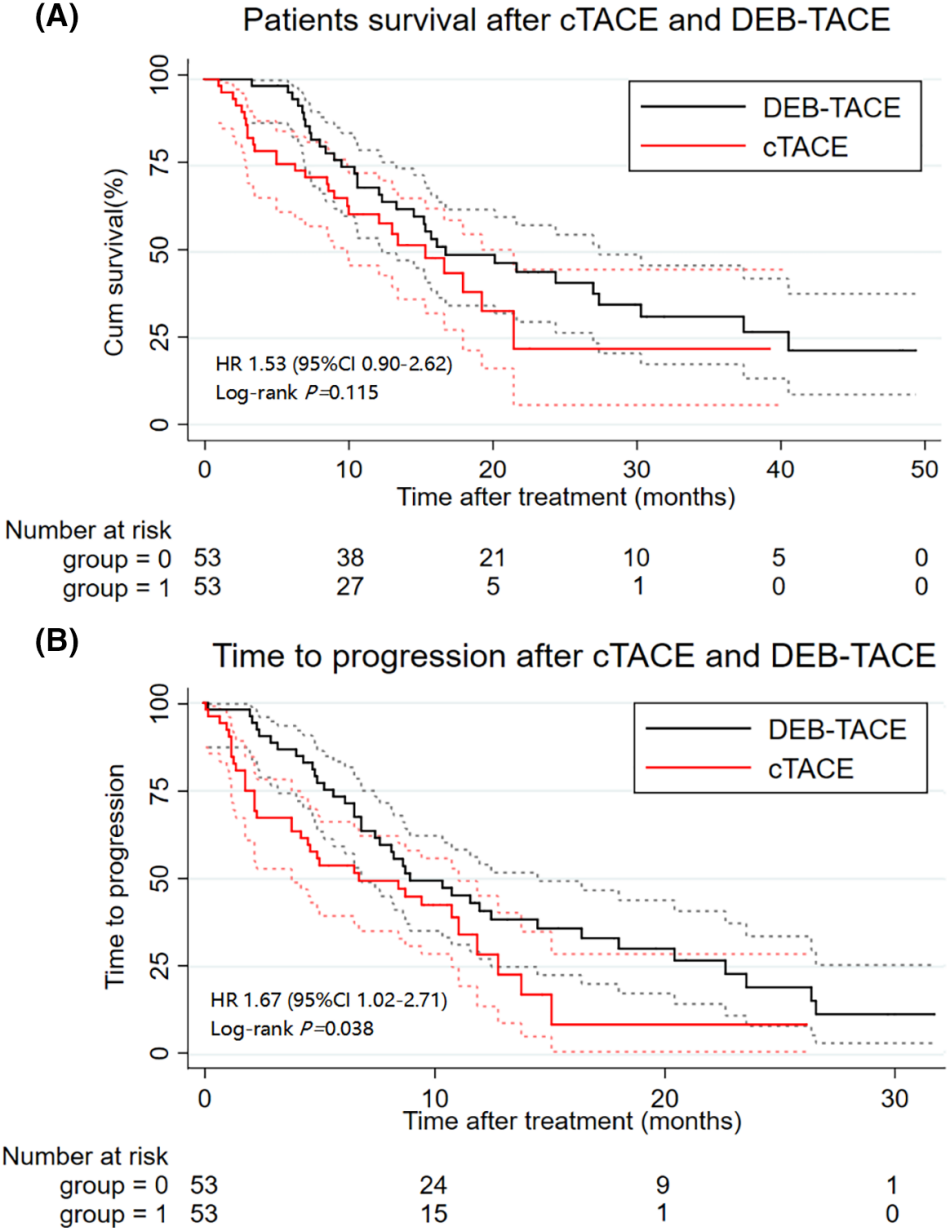
Overall survival (OS) and time to progression (TTP) between the DEB‐TACE group and cTACE group after propensity score‐matching analysis. (A) DEB‐TACE achieved comparable median OS compared with cTACE (16.7 vs 15.3 months; *p* = 0.115); (B) DEB‐TACE achieved longer median TTP compared with cTACE (8.9 vs. 6.7 months; *p* = 0.038). Comparison between groups was determined by log‐rank test. A value of *p* < 0.05 was considered significant.

Among the cohorts after PSM, 61 patients died of hepatic failure (29, 27.4%), tumor progression (18, 17.0%), and other reasons (14, 13.2%). The median OS did not significantly differ between the DEB‐TACE and cTACE groups (16.7 months vs. 15.3 months, *p* = 0.115; Figure 4A). However, the DEB‐TACE group had a longer median TTP (8.9 months, 95% confidence interval [CI]: 6.8, 14.4) than the cTACE group (6.7 months, 95% CI: 3.8, 11.0; *p* = 0.038; Figure 4B). The survival outcomes and treatment responses of patients experiencing recurrence are presented in Tables S1 and S2, respectively.

### 3.3 Complications and liver toxicity

Tables 3 and Table 4 summarize adverse events within 7 days and serious adverse events within 30 days in the two groups, respectively. No treatment‐related mortality occurred within 30 days. Supplementary post hoc analysis revealed that incidence rates of fever, abdominal pain, and vomiting within 7 days of the first TACE procedure were similar between the DEB‐TACE and cTACE groups. The mean increments in the TBil (12.7 umol/L vs. 29.4 umol/L, *p* = 0.006), ALBI score (0.243 vs. 0.383, *p* = 0.029), and ALT (92.1 U/L vs. 128.5 U/L, *p* = 0.050) within 7 days of the first TACE procedure were lower in the DEB‐TACE group than those in the cTACE group (Table 3 and Figure S1).

**TABLE 3:** Incidence of adverse events within 7 days of the first TACE between patients in the DEB‐TACE group and cTACE group after successful drainage (after propensity score matching).

Outcome (Number of patients)	Number (%)/Median (IQR)^a^			*p* Value
	Total (*n* = 106)	DEB‐TACE Group (*n* = 53)	cTACE Group (*n* = 53)	
Fever^b^	56 (52.8%)	26 (49.1%)	30 (56.6%)	0.560
Grade 1	39 (36.8%)	18 (34.0%)	24 (45.3%)	
Grade 2	14 (13.2%)	5 (9.4%)	9 (17.0%)	
Grade 3	2 (1.9%)	0	2 (3.8%)	
Grade 4	1 (0.9%)	0	1 (1.9%)	
Abdominal pain^c^	64 (60.4%)	30 (56.6%)	34 (64.2%)	0.136
Grade 1	40 (37.7%)	19 (35.8%)	21 (39.6%)	
Grade 2	20 (18.9%)	9 (17.0%)	11 (20.8%)	
Grade 3	4 (3.8%)	2 (3.8%)	2 (3.8%)	
Vomiting^b^	46 (38.7%)	21 (36.8%)	25 (40.3%)	0.711
Grade 1	28 (23.5%)	15 (26.3%)	13 (21.0%)	
Grade 2	15 (12.6%)	5 (8.8%)	10 (16.1%)	
Grade 3	3 (2.5%)	1 (1.8%)	2 (3.2%)	
Grade 4	0	0	0	
TBIL increment (umol/L)	21.1 (15.0–27.2)	12.7 (6.21–19.3)	29.4 (19.5–39.4)	0.006
ALB decrement (g/L)	3.1 (2.3–3.8)	2.4 (1.0–3.7)	3.8 (3.3–4.3)	0.057
ALBI score increment	0.313 (0.249–0.377)	0.243 (0.125–0.360)	0.383 (0.334–0.432)	0.029
ALT increment (U/L)	109.8 (91.5–128.1)	92.1 (65.9–118.3)	128.5 (103.0–153.9)	0.050

Abbreviations: ALB, albumin; ALBI, albumin‐bilirubin; ALT, alanine aminotransferase; cTACE, conventional transarterial chemoembolization; DEB‐TACE, drug‐eluting beads transarterial chemoembolization; TBil, total bilirubin.

^**a**^ Median with interquartile range is shown for quantitative variables, whereas counts with proportions are shown for categorical variables.

^b^ No Grade 5 patients recorded.

^c^ Only Grade 1 to 3 according to the Common Terminology Criteria of Adverse Events v4.0.

**TABLE 4:** Incidence of serious adverse events within 30 days of the first TACE between patients in the DEB‐TACE group and cTACE group after successful drainage (after propensity score matching).

Outcome (Number of patients)	Total (*n* = 106)	DEB‐TACE Group (*n* = 53)	cTACE Group (*n* = 53)	*p* value
Myelosuppression	10 (9.4%)	4 (7.5%)	6 (11.3%)	0.741
Grade 3	8 (7.5%)	3 (5.7%)	5 (9.4%)	
Grade 4	2 (1.9%)	1 (1.9%)	1 (1.9%)	
Hepatic failure	14 (13.2%)	3 (5.7%)	11 (20.8%)	0.045
Grade 3	11 (10.4%)	3 (5.7%)	8 (15.1%)	
Grade 4	3 (2.8%)	0	3 (5.7%)	
ALT increase	19 (17.9%)	4 (7.5%)	15 (28.3%)	0.011
Grade 3	19 (17.9%)	4 (7.5%)	15 (28.3%)	
Grade 4	0	0	0	
TBil increase	24 (22.6%)	6 (11.3%)	18 (34.0%)	0.005
Grade 3	23 (21.7%)	6 (11.3%)	17 (32.1%)	
Grade 4	1 (0.9%)	0	1 (1.9%)	
Liver abscess	6 (5.7%)	2 (3.8%)	4 (7.6%)	0.401

Abbreviations: ALT, alanine aminotransferase; cTACE, conventional transarterial chemoembolization; DEB‐TACE, drug‐eluting beads transarterial chemoembolization; TBil, total bilirubin.

Major serious adverse events included myelosuppression, hepatic failure, and elevated ALT and TBil levels. The DEB‐TACE group showed lower incidence rates of hepatic failure (5.7% vs. 20.8%, *p* = 0.045) and elevated ALT and TBil levels (7.5% vs. 28.3%, *p* = 0.011; 11.3% vs. 34.0%, *p* = 0.005, respectively) within 30 days than the cTACE group (Table 4 **)**.

### 3.4 Univariable and multivariable analyses

In the univariable analysis, AFP ≥200 ng/mL (*p* = 0.031), elevated pre‐TACE TBil (*p* = 0.006), multiple intrahepatic tumors (*p* = 0.003), presence of PVTT (*p* = 0.033), and extrahepatic metastasis (*p* = 0.002) were significant prognostic factors of poor OS. Elevated pre‐TACE TBil level (*p* = 0.028), multiple intrahepatic tumors (*p* = 0.005), presence of PVTT (*p* = 0.029), extrahepatic metastasis (*p* = 0.001), and cTACE treatment (*p* = 0.041) were significant prognostic factors of poor TTP (Table 5).

**TABLE 5:** Univariate and multivariate analyses of predictors of overall survival after treatment (after propensity score matching).

	Overall survival				Time to progression			
	Univariate	Multivariate			Univariate	Multivariate		
Factor	*p* Value	HR	95% CI	*p* Value	*p* Value	HR	95% CI	*p* Value
Age	0.293				0.192			
Sex	0.405				0.343			
HBV	0.342				0.356			
AFP, <200 vs ≥200 ng/mL	0.031	1.45	0.85–2.46	0.065	0.052			
TBil on adimission (per 1 umol/L increase)	0.138				0.457			
Pre‐TACE TBil^a^ (per 1 umol/L increase)	0.006	1.01	1.00–1.01	0.039	0.028	1.00	0.99–1.01	0.067
ALBI score	0.242				0.147			
Intrahepatic tumors number, 1 vs. ≥2	0.003	1.87	1.11–3.17	0.019	0.005	1.94	1.21–3.10	0.006
Tumor size^b^	0.062				0.176			
PVTT	0.033	1.74	1.01–3.00	0.046	0.029	1.83	1.13–2.97	0.014
Extrahepatic metastasis	0.002	2.09	1.21–3.60	0.008	0.001	1.97	1.20–3.24	0.007
Treatment allocation^c^	0.119				0.041	1.68	1.02–2.78	0.042
BCLC stage	0.133				0.120			

Abbreviations: AFP, alpha‐fetoprotein; BCLC, Barcelona Clinic Liver Cancer; PVTT, portal vein tumor thrombus; TBil, total bilirubin.

^a^ Pre‐TACE TBil, TBil measured after successful drainage before performing TACE.

^b^ Tumor size, size of the largest tumor.

^c^ Treatment allocation, conventional transarterial chemoembolization or drug‐eluting beads transarterial chemoembolization.

In the multivariable analysis, elevated pre‐TACE TBil (*p* = 0.039), multiple intrahepatic tumors (*p* = 0.019), presence of PVTT (*p* = 0.046), and extrahepatic metastasis (*p* = 0.008) were independent factors of poor OS (hazard ratios, 1.01, 1.87, 1.74, and 2.09, respectively). Multiple intrahepatic tumors (*p* = 0.006), presence of PVTT (*p* = 0.014), extrahepatic metastasis (*p* = 0.007), and cTACE treatment (*p* = 0.042) remained independent predictors of poor TTP (hazard ratios, 1.94, 1.83, 1.97, and 1.68, respectively; Table 5).

### 3.5 Identification of the cutoff value of pre‐TACE TBil level for sequential TACE

The pre‐TACE TBil cutoff was set to 99.0 μmol/L, as analyzed by X‐tile, and patients were segregated into high and low TBil groups based on this value (Figure S2). A high pre‐TACE TBil (>99.0 μmol/L) at the first TACE procedure was associated with poor OS in both the DEB‐TACE (hazard ratio = 2.28, 95% CI: 1.37, 3.79; 26.9 months vs. 11.8 months, *p* = 0.001) (Figure S3A) and cTACE (hazard ratio = 2.96, 95% CI: 1.78, 4.91; 20.8 months vs. 7.3 months, *p* < 0.001) groups (Figure S3B). Analyzing data of patients with pre‐TACE TBil level >99.0 μmol/L using the Kaplan–Meier curve (Figure S4), we observed that the DEB‐TACE group had a higher OS than the cTACE group.

### 3.6 Subgroup analysis

**FIGURE 5:**
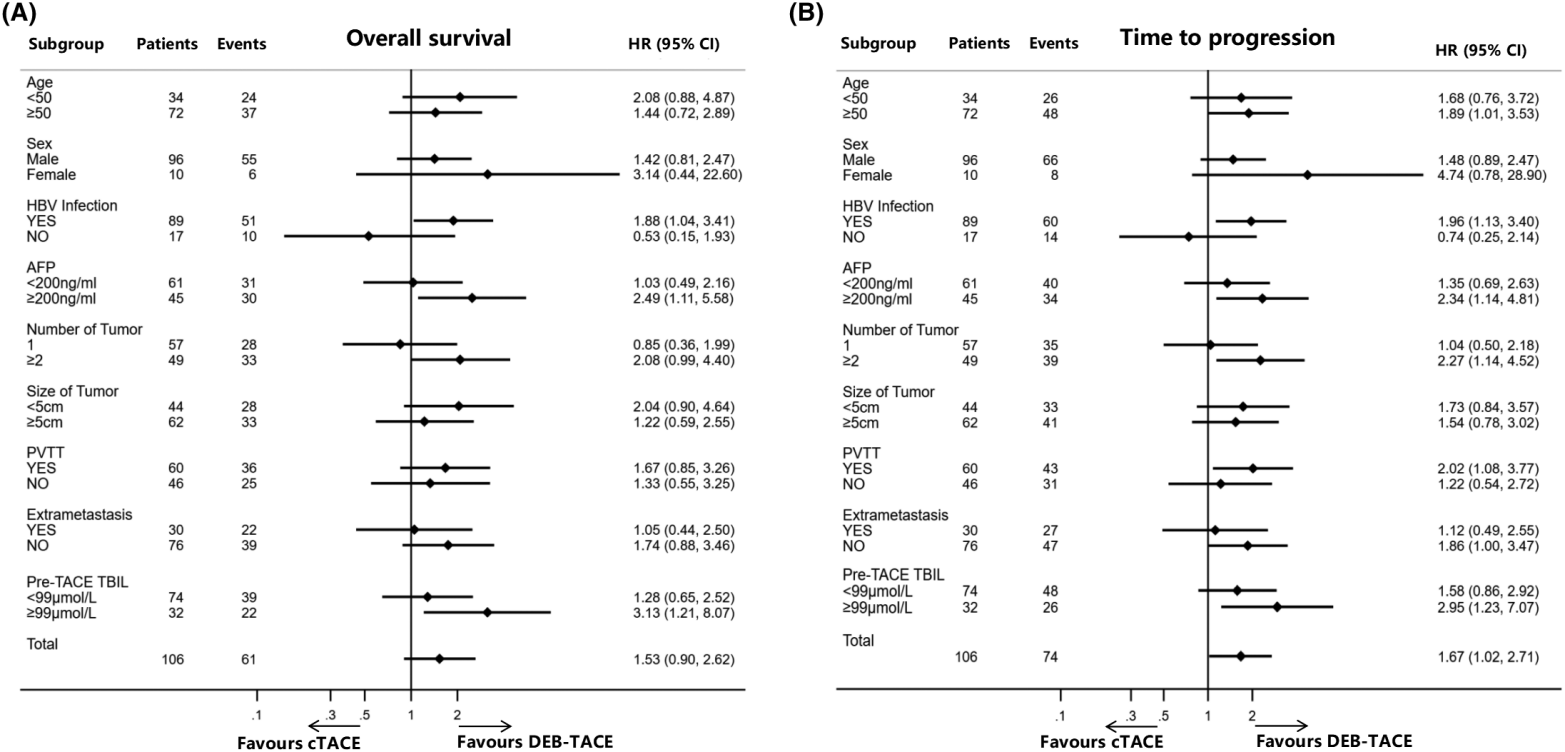
Forest plot of overall survival (OS) and time to progression (TTP) of patients between DEB‐TACE and cTACE groups. (A) OS; (B) TTP. AFP, alpha‐fetoprotein; CI, confidence interval; cTACE, conventional transarterial chemoembolization; DEB‐TACE, drug‐eluting beads transarterial chemoembolization; HBV, hepatitis B virus; HR, hazard ratio; PVTT, portal vein tumor thrombus; TBil, total bilirubin.

In the subgroup analysis, it was observed that among patients with hepatitis B virus infection, an AFP level ≥200 ng/mL, and a pre‐TACE TBil level >99 μmol/L, the DEB‐TACE group achieved superior OS compared to the cTACE group, as depicted in Figure 5A. Additionally, the DEB‐TACE group achieved enhanced TTP compared to the cTACE group, particularly among patients aged ≥50 years and those with characteristics such as hepatitis B virus infection, AFP levels ≥200 ng/mL, multiple intrahepatic tumors, PVTT, extrahepatic metastasis, and pre‐TACE TBil levels >99 μmol/L, as shown in Figure 5B.

## 4 DISCUSSION

To the best of our knowledge, this is the first study to compare the efficacy and safety of DEB‐TACE and cTACE after successful biliary drainage for treating unresectable HCC patients with B1‐BDI. Our findings were as follows^1^: DEB‐TACE had better results in TTP and local response than cTACE, although both modalities had similar OS^2^; DEB‐TACE caused milder liver injury than cTACE with comparable tolerability^3^; A TBil level below 99 μmol/L, achieved through successful biliary drainage before TACE, contributed to survival benefits^4^; DEB‐TACE could be a potential option even if the ideal pre‐TACE TBil cutoff level was not achieved after successful drainage.

cTACE reportedly affords a better prognosis than conservative management for unresectable HCC with BDI.^30^ DEB‐TACE led to better tumor response and lower impact on liver function than cTACE in patients with advanced‐stage HCC,^19^ indicating that DEB‐TACE can be an alternative for patients with unresectable HCC with B1‐BDI. However, few relevant studies have explored these modalities among this patient population. Thus, we conducted the current study and revealed that DEB‐TACE resulted in higher PR and ORR than cTACE. In the current study, the ORRs of the two groups (64.2% vs. 39.6%) were similar to those of HCC patients with more advanced disease in the PRECISION V study (52.4% vs. 34.7%).^19^ In addition, we observed that DEB‐TACE resulted in a longer TTP than cTACE (8.9 vs. 6.7 months, *p* = 0.038). These results can be explained as follows: DEB‐TACE was designed to overcome the limitations of cTACE by persistently and stably releasing chemotherapy drugs and effectively occluding tumor blood supply.^31^ This results in lower drug escape, higher drug concentration at the target lesion, and more effective tumor cell killing, which improves tumor response and prolongs TTP in patients with HCC. In multivariate analysis, DEB‐TACE independently predicted a prolonged TTP (hazard ratio = 1.68), further supporting our results. However, the better response to treatment and longer TTP observed with DEB‐TACE did not translate into significantly improved survival with respect to cTACE, although a trend in favor of DEB‐TACE was observed (16.7 vs. 15.3 months, *p* = 0.115). Nevertheless, when compared with cTACE in the OS result of patients from the previous studies conducted by Lai et al. (13.4 months)^32^ and Feng et al. (13.0 months),^30^ DEB‐TACE in present study (16.7 months) still demonstrated potentially superior OS results. These results suggest that DEB‐TACE could be considered as a potential treatment option for patients with unresectable HCC presenting with B1‐BDI.

Regarding safety, we detected fewer post‐procedural increments in ALT levels, ALBI scores, and TBil values within 7 days and a lower frequency of serious hepatic adverse events within 30 days in the DEB‐TACE group. These results illustrated that post‐procedural liver toxicity was remarkably lower with acceptable tolerability in the patients who underwent DEB‐TACE than in those who underwent cTACE, consistent with the results of Liu et al.^33^ and the PRECISION V study.^19^ This may be attributed to the effects of the drug‐eluting beads, which minimized drug escape to normal liver tissue, allowing gradual drug release.^34^ Notably, post‐procedural fluctuating jaundice may be associated with TACE‐related complications, including hepatocellular injury, ischemic cholangitis, or necrotic debris floating into the bile duct,^30^, ^35^ thus warranting liver protection, antibiotic treatment, or percutaneous drainage for prompt interventions. DEB‐TACE has been associated with a higher rate of bile duct injury than cTACE, given that the peribiliary plexus of bile ducts, supplied by the hepatic arterial branches, can be damaged during embolization by the small bead agent.^36^ However, we noted a higher frequency of post‐procedural TBil increments in the cTACE group. This result can be attributed to the use of beads (100–300 or 300–500 μm) in our study, which are large enough not to travel deep into the peribiliary plexus, thereby causing minimal ischemic damage to the bile ducts.^37^ Additionally, effective biliary drainage before TACE can appropriately release high bile duct pressure and increase cholestatic liver tolerance to ischemia.^38^ Conversely, lipiodol used for cTACE can spread more distally than 100–300 μm beads from the sinusoids to the distal portal vein branches, facilitating dual (arterial and portal) embolization of the peri‐biliary plexus, thereby causing severer liver and biliary damage.^39^

Previously, extrahepatic metastasis, PT‐INR prolongation, and vascular invasion were found to be poor prognostic factors.^12^ In the current study, based on multivariate analysis, we identified multiple intrahepatic tumors, PVTT, extrahepatic metastasis, and high pre‐TACE TBil levels as negative prognostic factors, representing heavy tumor burden, worse disease severity, and poor liver function associated with poor prognosis.^40^ Hyperbilirubinemia is a relative contraindication for HCC chemoembolization, as it frequently represents a substantial risk of postoperative liver failure.^5^ However, Choi et al.^12^ have concluded that obstructive hyperbilirubinemia in HCC patients with central BDI is not a contraindication for TACE, and TACE can be performed along with appropriate biliary intervention. Furthermore, Park et al.^41^ have reported that biliary drainage to normalize the TBil level before chemoembolization may not be mandatory in HCC patients with BDI, and only 51%–65% of HCC patients with obstructive jaundice can achieve successful bile drainage following PTCD and ERCP. HCC patients with BDI appeared to have advanced BCLC stages and Child‐Pugh classes at diagnosis owing to the high tumor burden and hyperbilirubinemia,^12^, ^21^ resulting in most patients receiving insufficient treatment according to the present guideline. Based on our Cox analysis, neither the BCLC stage nor ALBI score were deemed independent prognostic factors (all *p >* 0.05), further implying that these systems may be unsuitable for the examined patient population.^12^ Furthermore, HCC patients with BDI often struggle to meet the Child‐Pugh class A requirements for most antitumor therapies due to the presence of jaundice. This palliative drainage may consequently prolong waiting periods for anticancer therapy. In clinical practice, no standard timing for performing the antitumor treatment after drainage has been established. Therefore, in our study, TACE was performed after successful drainage was confirmed and was allowed by the patient's general condition. Ultimately, a pre‐TACE TBil level of 99 μmol/L represented an optimal threshold for guiding subsequent TACE following successful biliary drainage, effectively distinguishing patients into two distinct survival groups. This result further confirmed that hyperbilirubinemia in HCC patients with BDI is mainly caused by obstructive jaundice rather than hepatic insufficiency or tumor progression.^41^, ^42^, ^43^ Thus, patients may have sufficient liver function to tolerate aggressive treatment. Moreover, our post hoc comparison revealed that patients with a pre‐TACE TBil level >99 μmol/L achieved greater benefits with DEB‐TACE than with cTACE. Hence, DEB‐TACE may be a potential local treatment option when TBil does not rapidly return to normal levels after drainage, given that DEB‐TACE can rapidly reduce the tumor burden without severely damaging liver function.

This study has some limitations. First, we only focused on patients with unresectable HCC with B1‐type BDI, limiting the generalizability to other types of BDI. Second, there was no standard pre‐TACE TBil level for sequential TACE after effective biliary drainage. TACE was performed when the bilirubin level was as low as possible, which may be a subjective approach, resulting in a deviation from the optimal pre‐TACE TBil cutoff value. Thus, the cutoff value should be externally validated. We plan to continue searching for a specific tool to comprehensively assess HCC patients with B1‐BDI and identify optimal candidates for sequential TACE in the future. Moreover, this study used a retrospective design; hence, the risk of confounding bias between the two groups cannot be ignored, although PSM was performed to balance baseline characteristics. Further prospective randomized controlled investigations are needed.

In summary, after successful biliary drainage, DEB‐TACE might be a potential therapeutic option for unresectable HCC patients with B1‐type BDI, and a TBil level of 99 μmol/L can be used as an upper limit value for subsequent TACE.

## AUTHOR CONTRIBUTIONS

**Wenzhe Fan:** Conceptualization (equal); data curation (equal); formal analysis (equal); funding acquisition (equal); methodology (equal); resources (equal); supervision (equal); writing – original draft (equal); writing – review and editing (equal). **Xinlin Zheng:** Data curation (equal); formal analysis (equal); methodology (equal); validation (equal); visualization (equal); writing – original draft (equal); writing – review and editing (equal). **Xiao Zhao:** Data curation (equal); formal analysis (equal); methodology (equal); validation (equal). **Bowen Zhu:** Data curation (equal); formal analysis (equal); methodology (equal). **Yanqin Wu:** Data curation (equal); methodology (equal); validation (equal). **Miao Xue:** Data curation (equal). **Rong Tang:** Data curation (equal). **Zhen Huang:** Data curation (equal). **Liangliang Qiao:** Data curation (equal). **Mingjian Lu:** Data curation (equal). **Yiyang Tang:** Formal analysis (equal); methodology (equal). **Jian Wu:** Funding acquisition (equal); project administration (equal); resources (equal); supervision (equal). **Jiaping Li:** Conceptualization (equal); funding acquisition (equal); project administration (equal); resources (equal); supervision (equal).

## CONFLICT OF INTEREST STATEMENT

The authors have no conflicts of interest to declare.

## ETHICS STATEMENT

The study was approved by the Institutional Review Board of the Sun Yat‐Sen University the First Affiliated Hospital, and the need for informed consent was waived owing to the retrospective nature of the study. All procedures involving human participants were conducted in accordance with the Declaration of Helsinki.

## Supporting information


Appendix S1.

## Data Availability

The datasets used in this study are available from the corresponding author upon reasonable request.
