# Supplementary material for: DEB‐TACE versus cTACE for unresectable HCC with B1‐type bile duct invasion after successful biliary drainage: A propensity score matching analysis
Source: Cancer Med. 2024 Jul 6;13(13):e7419. doi: 10.1002/cam4.7419 (PMC11226754; doi:10.1002/cam4.7419)
Supplement: Supplementary file 1 — Appendix S1. [file CAM4-13-e7419-s001.docx]

**Supplemental Materials**

**
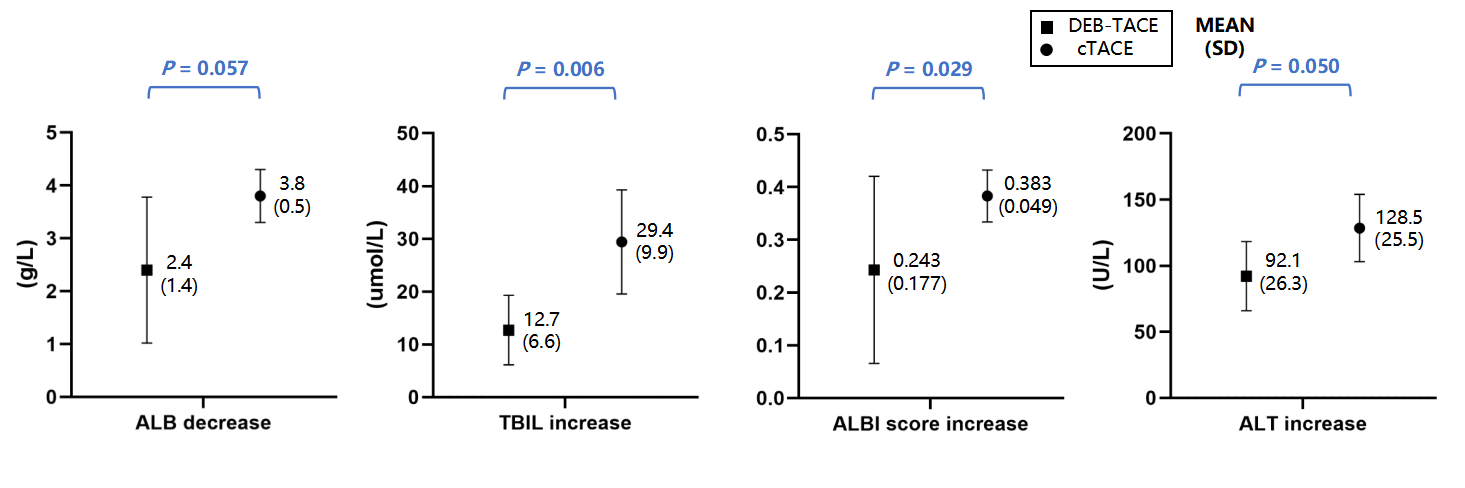
**

**Figure S1. The comparison of changes in ALB, TBil, ALBI score and ALT after TACE with 1 week between the DEB-TACE and cTACE groups.**

Abbreviations: *ALB*, albumin; *ALBI*, albumin-bilirubin; *ALT*, alanine aminotransferase; *cTACE*, conventional transarterial chemoembolizatio; *DEB-TACE*, drug-eluting beads transarterial chemoembolization; *TBil*, total bilirubin.


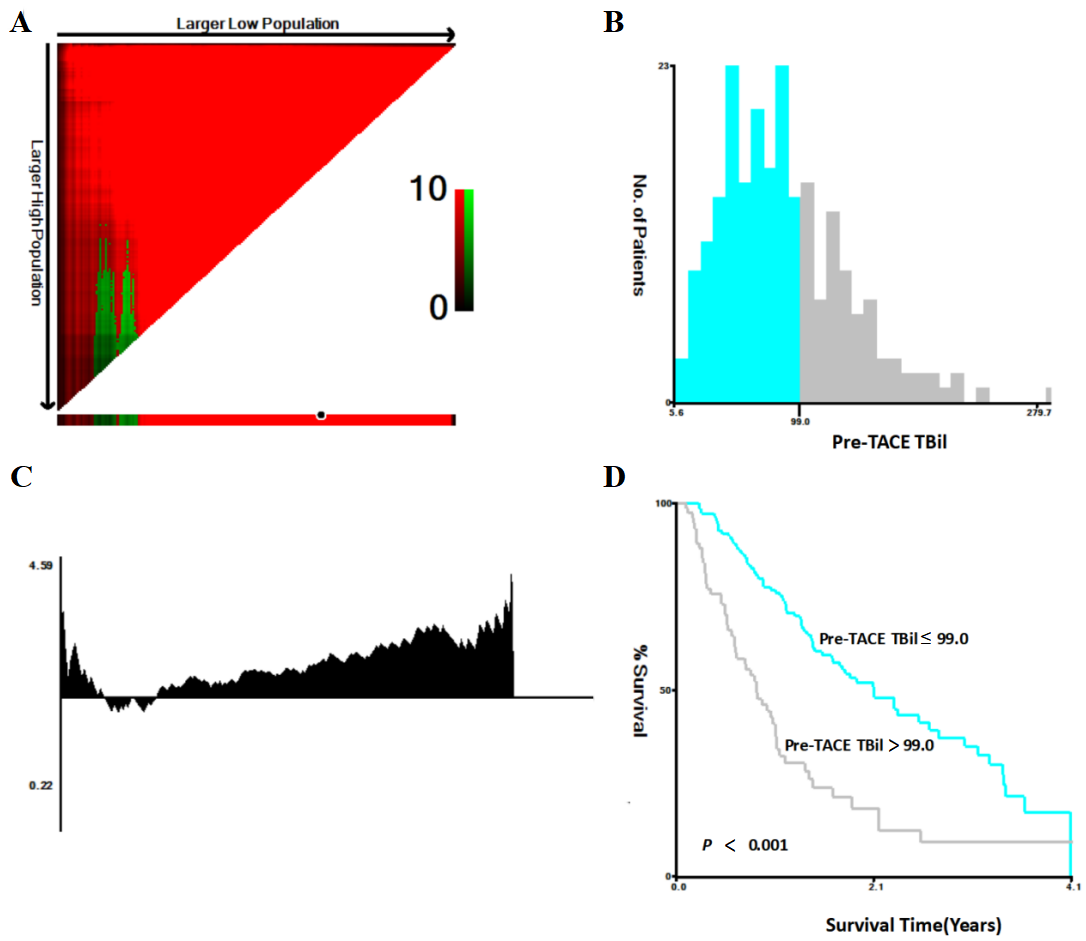


**Figure S2. X-tile plots of the pre-TACE TBil from all included patients (before PSM) based on overall survival. The plot shows the χ2 log-rank values that were created when the cohort was divided into two populations. The cutoff point, which is highlighted by a black/ white circle (A), is demonstrated on a histogram of the entire cohort (B), the relative risks for all cutoff points from low to high (left to right, x-axis), are calculated as event in high population/event risk in low population (C), a Kaplan-Meier overall survival curve (D). The pre-TACE TBil was divided at the optimal cutoff point, as defined by the most significant point on the plot (>99.0 and ≤99.0 µmol/L, *P*<0.001).**


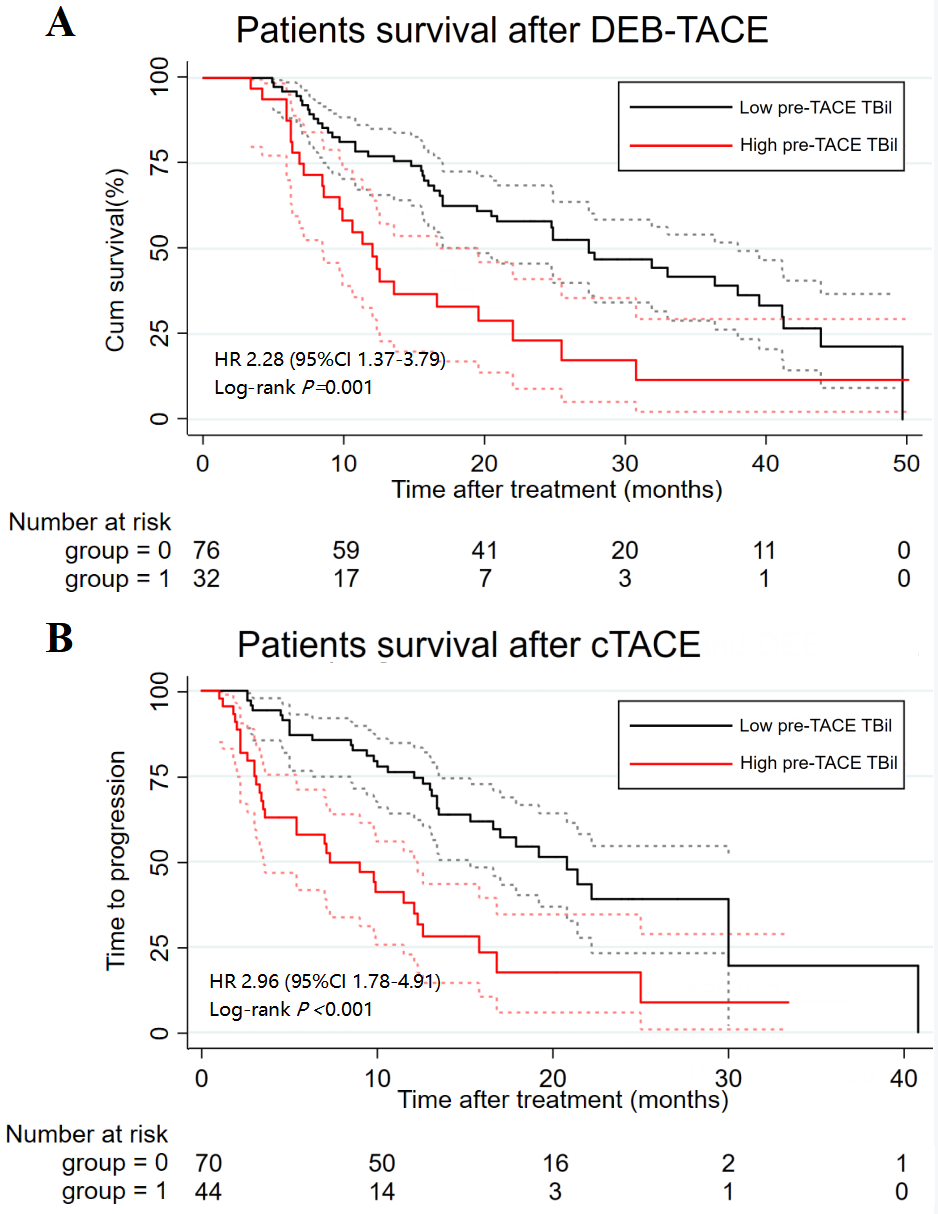


**Figure S3. The overall survival analysis of HCC patients with B1-BDI. The Kaplan-Meier curve analysis demonstrated high overall survival rates for the patients presenting with a lower pre-TACE TBil level (≤99.0 µmol/L) in both DEB-TACE group (A) and cTACE group (B).**

**
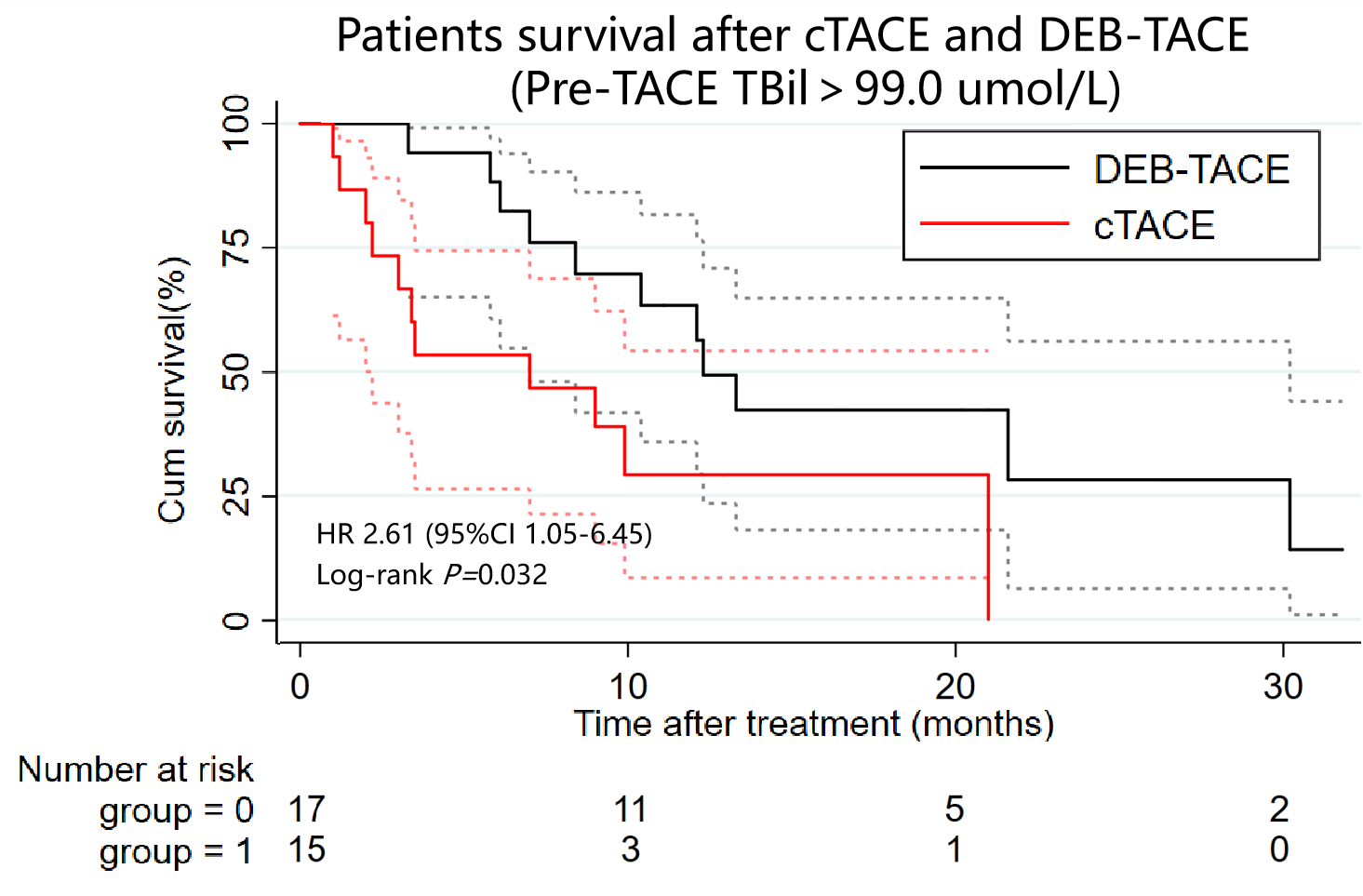
Figure S4. The overall survival analysis of patients with pre-TACE TBil >99.0 µmol/L after TACE. The Kaplan-Meier curve analysis demonstrated higher overall survival rates for the patients receiving DEB-TACE than cTACE.**

**Table S1. Survival outcomes of different treatment allocations with different recurrence status.**

| **Cohort** | **Recurrence (number)** | **Median OS (month) (95% CI)** | ***P* value** |
| --- | --- | --- | --- |
| DEB-TACE | Absence (10) | NA^†^ | <0.001 |
|  | Presence (35) | 13.3 (9.5-16.7) |  |
| cTACE | Absence (13) | NA^†^ (15.3-NA^†^) | <0.001 |
|  | Presence (24) | 9.9 (6.3-16.6) |  |

CI, confidence interval

^†^NA, no patient deaths occurred during the observation period.

**Table S2. Tumor response of different treatment allocations with or without recurrence.**

| **Variable** | **Group (N, %)** | | | | | |
| --- | --- | --- | --- | --- | --- | --- |
|  | **DEB-TACE** | | | **cTACE** | | |
|  | **Without recurrence**  **(N=10)** | **With recurrence**  **(N=35)** | ***P* value** | **Without recurrence**  **(N=13)** | **With**  **recurrence**  **(N=24)** | ***P* value** |
| Complete response | 2 (20%) | 2 (5.7%) | 0.209 | 1(7.7%) | 1 (4.2%) | 0.651 |
| Partial response | 8 (80%) | 22 (62.9%) | 0.310 | 8 (61.5%) | 11 (45.8%) | 0.362 |
| Objective response | 10 (100%) | 24 (68.8%) | 0.060 | 9 (50%) | 12 (50%) | 0.260 |
| Stable disease | 0 | 11 (31.4%) | 0.089 | 4 (30.8%) | 12 (50%) | 0.315 |

Abbreviations: *cTACE*, conventional transarterial chemoembolization; *DEB-TACE*, drug-eluting beads transarterial chemoembolization.
